# Preparedness of district clinical laboratories towards ISO 15189: 2012 accreditation scheme in Lake Zone, Tanzania (a descriptive cross-sectional study)

**DOI:** 10.11604/pamj.2022.41.208.25692

**Published:** 2022-03-14

**Authors:** Betrand Msemwa, Vitus Silago, Conjester Isdory Mtemisika, Nabina Simeo Golola, Martha Fidelis Mushi

**Affiliations:** 1 Institute of Health and Allied Sciences, Catholic University of Health and Allied Sciences, Mwanza, Tanzania,; 2 Department of Microbiology and Immunology, Catholic University of Health and Allied Sciences, Mwanza, Tanzania,; 3 Central Pathology Laboratory, Bugando Medical Centre, Mwanza, Tanzania

**Keywords:** ISO 15189:2012 standards, laboratory accreditation schemes, Tanzania

## Abstract

**Introduction:**

diagnosis, prevention, and surveillance of diseases relies on high quality laboratory services. However, accessibility and availability of the quality laboratory services among healthcare facilities remains a serious challenge among sub-Saharan African countries. This study investigated the preparedness of district clinical laboratories for ISO 15189: 2012 accreditation scheme using Stepwise Laboratory Quality Improvement Process towards Accreditation (SLIPTA) checklistin Lake Zone, Tanzania.

**Methods:**

this descriptive cross-sectional study was conducted from September 2018 to August 2019 among clinical laboratories at district hospitals and council designated hospitals in Lake Zone regions of Tanzania. Data were collected using the verified WHO-SLIPTA checklist. In each laboratory, either the laboratory manager, quality officer or safety officer was interviewed.

**Results:**

a total of 10 health laboratories affiliated to 6 districts and 4 councils designated hospitals were enrolled. Six laboratory managers and four quality officers were interviewed. Six (60%) and 4 (40%) laboratories were under government ownership and private ownership, respectively. The majority (70%, n=7) of medical district laboratories in Lake Zone-Tanzania were not registered for WHO-SLIPTA.

**Conclusion:**

about two third of district and council designated hospital laboratories in Lake zone are not registered for WHO-SLIPTA indicating unpreparedness towards ISO 15189: 2012 accreditation.

## Introduction

District hospital laboratories in Tanzania are the third level of health services closer to the community and are intended to provide basic health diagnostic services which include Parasitology, Hematology and Blood transfusion, Microbiology and Clinical Chemistry. The quality of diagnostic services provided in the laboratory pre-determines the quality of health care provided in the facility. Well-functioning health systems at district healthcare level are essential and fundamental in provision of laboratory services which are efficient, reliable and reproducible [1]. Patients care in diagnosis, prevention, surveillance and investigations of diseases outbreaks are centered in laboratories [2]. In sub-Saharan African countries like Tanzania, accessibility and availability of the quality laboratory services in healthcare settings remains a serious challenge [3,4]. This called for a need of strengthening the quality of the laboratory services through different accreditation schemes.

Southern African Development Community Accreditation Service (SADCAS) a regional accreditation scheme established in 2007 and Laboratory Accreditation Program (LAP) offered by College of American Pathologists (CAP) an international accreditation scheme developed in 1961, are examples of accreditation schemes developed for improvement of laboratory performance to meet the international standards [1,5]. These accreditations schemes are important to upholds quality of laboratory services and improve laboratory performance to the international standards [1,5]. For easy adaptation of accreditation, World Health Organization (WHO) regional management in Africa region introduced Strengthening Laboratory Management towards Accreditation (SLMTA) and Stepwise Laboratory Quality Improvement Process towards Accreditation (SLIPTA) programs [1,5]. The programs aim not to replace International Organization for Standards (ISO) 15189 which aims to improve quality management system and competence of medical laboratories to meet international standards. But rather to guide pathway towards enrollment to full ISO 15189 schemes through measuring, monitoring and recognizing improved quality of health laboratory services.

In the SLIPTA program, the laboratories are assessed in three parts which include laboratory profile, laboratory audits and summary of audit findings [6]. Under this program, laboratory services and management system are assessed and recognized on star's ascending scale from 0 to 5 stars. Scores of laboratory systems after assessment are: less than 55% graded as no star, 55%-64% graded as 1 star, 65%-74% graded as 2 stars, 75%-84% graded as 3 stars, 85%-94% graded as 4 stars and above 95% graded as 5 stars [1,6]. After attaining a specific star, laboratories are required to maintain the status while working hard to attain the next star level. Any health laboratory which will be awarded 5 stars will be motivated to enroll in an established international or regional ISO 15189 accreditation scheme [1,6,7].

In 2014, the Ministry of Health-Tanzania with the support and assistance of US Centers for Disease Control and Prevention (CDC) and American Society for Laboratory Medicine (ASLM) invested in trainings to support medical laboratories´ accreditation schemes viz. ISO 15189 through the WHO-SLIPTA checklist [8]. However, the achievement of the ISO 15189 accreditation schemes depends much on the health laboratory management system and staffs commitment. In district hospitals of Lake Zone of Tanzania, little is known regarding the preparedness of clinical laboratories towards ISO 15189: 2012 accreditation schemes. This is known to have an effect on the success of the ISO 15189 accreditation schemes and can increase the associated costs of accreditations processes. Therefore, the main objective of this study was to investigate the preparedness of district clinical laboratories towards ISO 15189: 2012 accreditation scheme through WHO-SLIPTA schemes in Lake Zone, Tanzania.

## Methods

**Study design, duration, setting and participants:** a descriptive cross-sectional study was conducted from September 2018 to August 2019 in 10 clinical laboratories affiliated to district hospitals and council designated hospitals located in four Lake Zone regions named Shinyanga, Kagera, Mara and Mwanza. A simple random sampling technique was used to select 10 laboratories for this study. SLIPTA checklist was used for observation of laboratories and interviewing of study participants who included laboratory managers or quality officers or safety officers representing their laboratories. The SLIPTA checklist was sectioned into (i) laboratory profile, (ii) infrastructures (buildings, power/electricity supply, water supply and fire extinguishers), (iii) machinery, (iv) reagents supply, (v) personnel safety, (vi) documentations and (vii) registered to SLIPTA scheme, attained and sustained star(s) ratings.

**Data analysis:** data analysis was done by using STATA software version 13 according to the study objectives. Results were presented into percentages/proportions for categorical variables and median (IQR)/mean (STD) for continuous variables.

**Ethical considerations:** this study was ethically cleared by the joint CUHAS/BMC Research Ethics and Review Committee with certificate number: CREC 301/2018. Laboratory related information e.g., names were stored anonymously using codes to ensure confidentiality.

## Results

**Laboratory profiles:** six laboratory managers and four quality officers from 10 clinical laboratories affiliated to district and council designated hospitals were interviewed. A total of 6 (60%), 3(30%) and 1(10%) laboratories were under government ownership, faith-based ownership and non-faith-based ownership, respectively. The studied laboratories had a total of 71 employees, with a median number of 7 [5-9] employees per laboratory. The academic qualifications of these employees ranged from Laboratory Scientist 3(4.2%), Laboratory Technologist38 (53.5%), Assistant Laboratory Technologist 30 (42.3%) to Laboratory attendants 15 (21.1%) (Table 1).

**Table 1:** laboratories´ profiles and infrastructures

Variables		Frequency (n)	Percentages (%)
**Laboratory profiles**	Median (IQR*) number of workers		
	Ownerships	7(5-9)	-
	Government	6	60
	Privates	4	40
	Private ownerships		
	Faith based ownerships	3	75
	Non-faith-based ownerships	1	25
**Infrastructures**	**Satisfaction by infrastructures**		
	Excellent	1	10
	Very good	1	10
	Good	5	50
	Poor	3	30
	Presence of standby generator		
	Present	9	90
	Absent	1	10
	**Alternative source of water**		
	Present	10	100
	Absent	0	0
	**Fire extinguishers**		
	Present	10	100
	Absent	0	0

* Interquartile range

**Laboratories´ infrastructures:** fifty percent (5/10) of the interviewees reported being satisfied with laboratory building and laboratory design. The majority of interviewees reported presence of standby generators (90%, n=9) during power cut off, presence of another source of clean water (100%, n=10) during water cut off and presence of fire extinguishers (100%, n=10) to fight fire outbreak (Table 1).

**Machinery:** a total of 8(80%) laboratories reported occurrences of analyzers´ breakdown, mostly (87.5%, 7/8) due to lack of planned preventive maintenance. Six out of 8 laboratories with analyzers´ breakdown did not have any analyzers´ backups, therefore during breakdowns they either stop collection and processing of samples (50%, n=3) or refer patients to nearby laboratories (50%, n=3) (Table 1).

**Reagents supply:** all (100%, n=10) laboratories reported incidences of reagents out of stocks of duration up to one month (90%, n=9). The common reason reported for reagents out of stocks was late supply (70%, n=7) from agencies, of which the majority of agencies (50%, n=5) took about 1 month to supply the ordered reagents (Table 1).

**Personnel safety:** the majority of laboratories (90%, n=9) reported sufficient availability of personal protective equipment´s (PPEs). On the other hand, all (100%) laboratories reported presence of official forms for needle stick injuries and HIV post exposure prophylaxis, however only 50% (5/10) of laboratories reported on the presence of official HBV vaccination programs. Interviewed laboratories reported presence of safety training programs, eye washing stations in case of splashes and proper waste segregation 60%, 30% and 80% respectively. Only 50% (5/10) of laboratories had warning signs, e.g., biohazard and inflammables on places (Table 2).

**Table 2:** laboratory machinery, reagents supplies, personnel safety, documentations and Status of SLIPTA registration

Variables		Frequency (n)	Percentages (%)
**Machinery**	**Analyzer breakdown**		
	Yes	8	80
	No	2	20
	**Reasons for analyzers breakdown**		
	Lack of maintenance	7	87.5
	Overload	1	12.5
	**Analyzer backups**		
	Yes	2	25
	No	6	75
	**Measures during breakdown**		
	Stop sample collection	3	50
	Refer patients to another lab	3	50
**Laboratory reagents**	Reagents out of stock (OS*)		
	Yes	10	100
	No	0	0
	**Duration for OS**		
	1 month	9	90
	3 months	1	10
	**Reasons for OS**		
	Late supply	7	70
	Late ordering	2	20
	Insufficient funds	1	10
	**Duration from ordering to supply**		
	Weeks	3	30
	1 month	5	50
	3 months	2	20
**Personnel safety**	**Personal protective equipments**		
	Present	9	90
	Absent	1	10
	HBV** vaccination program		
	Present	5	50
	Absent	5	50
	**Documentation for needle stick injuries**		
	Present	10	100
	Absent	0	0
	HIV*** prophylaxis		
	Present	10	100
	Absent	0	0
	**Safety training**		
	Present	6	60
	Absent	4	40
	**Emergence eye washing station**		
	Present	3	30
	Absent	7	70
	**Proper waste segregation**		
	Yes	8	80
	No	2	20
SLIPTA**** star(s)	**Status**		
	Registered with stars	3	30
	Unregistered	7	70

* out of stock **Hepatitis B virus ***Human immunodeficiency virus ****Stepwise Laboratory Quality Improvement Process towards Accreditation

**Figure 1:**
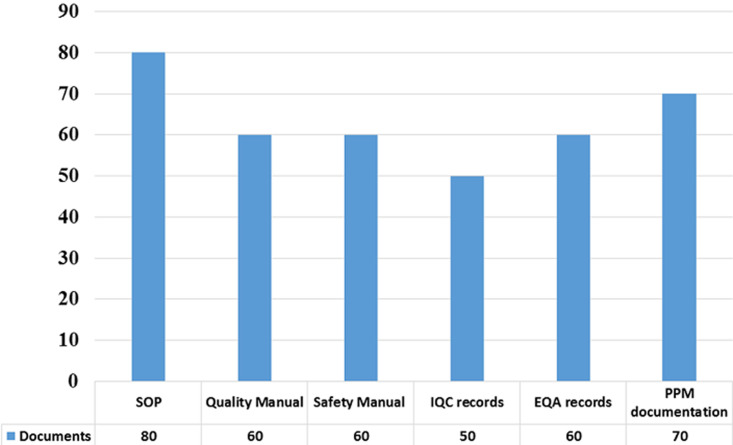
laboratory records and documentations

**Documentations:** standard operation procedures (SOPs), quality manuals, safety manuals, internal quality control (IQC) records, external quality assurance (EQA) records and planned preventive maintenance (PPM) documentations present among 80% (n=8), 60% (n=6), 60% (n=6), 50% (n=5), 60% (n=6) and 70% (n=7) of all laboratories (Figure 1).

**Registration and status of SLIPTA star(s) ratings:** during this study period, only three (30%, 3/10) laboratories were registered on WHO-SLIPTA systems and attained star(s) ratings. Of the laboratories with star(s), only one laboratory (33.3%, 1/3) attained 3 stars and the rest attained 1 star each (33.3%, 1/3) (Table 1 and Table 2).

## Discussion

The main objective of this study was to determine the preparedness of district clinical laboratories towards ISO 15189: 2012 accreditation schemes in Lake Zone, Tanzania. This study mainly focused on the laboratory´s capacity of delivering uninterrupted quality services by determining infrastructures, machinery, reagents supply, personnel safety and documentations. Nearly two third (60%) of laboratories enrolled in the study were government/public owned. The majority (75%) of private owned laboratories were under faith-based ownership. The median number of laboratory workers in studied laboratories align with “Health Sector Staffing Levels guideline 2014-2019”, which recommends a minimum of 3 Laboratory Technologists, 2 Assistant Laboratory Technologists and 4 Laboratory Attendants (2 Medical Attendants and 2 Mortuary Attendants) [9]. Although the “Health Sector Staffing Levels guideline 2014-2019” did not mention Laboratory Scientist as part of staff in district level laboratories, this study observed the presence of three Laboratory Scientists in three different private owned laboratories. However, only one laboratory with a Laboratory Scientist attained one star.

In this study, only 3 (30%) laboratory´s representatives reported to have poor infrastructures as long as laboratory architectural designs is concerned. Moreover, the vast number of laboratories were connected to standby generators (90%) to back-up power during cut-offs, alternative source of water supply (100%) to ensure continuous water supply and fire extinguishers (100%) to fight fire outbreaks. Having a standby generator as a back-up for power supply in time of emergencies as reported is the current study ensures constant running of machines and optimal storage of reagents. Furthermore, all investigated laboratories in the current study reported to have an alternative source of water. The optimal operations of laboratory and control of laboratory associated infections and contamination require constant flow of running water. Unstable power supply and infrequently running of water minimizes the accuracy and timely testing, therefore set a barrier to improvement towards accreditation as reported previous [10].

The majority (80%) of clinical laboratories enrolled reported machinery breakdown, of which the larger proportion of laboratories (75%) with machinery breakdown had no back-up machines to keep running during breakdown, whereby lack of planned preventive maintenance (87.5%) was the reason for breakdown. Lack of in-country expertise for servicing and maintaining the laboratory machinery has previous being reported as factor for laboratory machinery breakdown. The long duration of laboratory machine maintenance is being associated with high cost of hiring international expert [11]. Machinery break down impairs the income gain of a particular laboratory. Therefore, quality assurance authorities should ensure timely machinery maintenance and back-up in clinical laboratories to improve quality and constant supplying of better and quality laboratory services. A constant supply of health services is fundamental in all heath care centers. This is at most depending on the availability of materials, reagents and supply throughout the year. In the studied setting, all laboratories reported to have reagents out of stock at least for one month in a year. Majority of laboratories reported late supply of reagents from vendor as a reason of not having a constant supply of laboratory reagents. This observation was also reported previously by Zhang *et al*. [12]. The characteristic of Tanzanian government procurement chain which involve central ordering systems and post-delivery payments and private suppliers who are the middlemen between customers and manufacturers or wholesale companies, might be the reason for late supplying of orders from laboratories. The said procurement system may hinder the implementation of improvement of laboratory services as reported previous from the same region [10].

In the current study, concerning prevention of infections and healthcare associated hazards, most laboratories reported adequate availability of PPEs but the opposite for warning signs, safety training and emergence eyes washing stations. Warning signs such as “no entry, authorized personnel only”, “biohazard” and “flammable chemicals” are important for both laboratory personnel and other laboratory users [13,14]. They help minimize laboratory accidents, contamination and spreading of infectious microorganisms [13,14]. Safety training among laboratory personnel impacts potential knowledge to ensure they adhere with safe working environment to minimize laboratory accidents, contamination and spreading of infectious microorganisms from one client to another inside and outside laboratory premises [14,15]. The emergency eye washing station protects from ocular damage by chemicals e.g., corrosive and inflammable chemical contacts with eyes and ocular infections by splashes from potentially infectious biological specimens [16,17]. For post-exposure precautions, the majority of laboratories had registry and reporting forms for needle stick injury and HIV post-exposure prophylaxis program, however availability of vaccine for HBV was inadequate. Clinical laboratory workers as they are directly and/or indirectly exposed to risks of acquiring viral hepatitis from HBV infections, they are supposed to be vaccinated so as they can be protected against HBV which are cross-transmitted through contact with body fluids [18,19].

According to accreditation schemes, WHO SLIPTA and ISO 15189: 2012, a good functioning laboratory should have a clear documentation and should be guided by the qualified protocols such as quality manuals, IQA and EQA records. These documentations are important in monitoring the laboratory performance and credibility of results provided by a particular laboratory. However, in the current study, the number of laboratories with documentations especially quality manuals, safety manuals, IQC records and EQA records is unacceptable. Quality manuals clearly communicate the framework for meeting laboratory quality system requirements, while safety manuals communicate developed standards and policies that aims at keeping the working environment safe for laboratory users. In general, quality manuals aim at ensuring the acceptable quality of all services offered by particular laboratory and safety manuals aim at ensuring preventive measures for the spreading of infections and occurrences such as needle stick injuries are well observed and adhered. The main purpose of performing IQC is to ensure that the released laboratory services particularly clients´ results are reliable and valid through detection, reduction and correction of any error in the laboratory´s internal analytical process. EQA aims at ensuring the effectiveness of a particular laboratory´s quality management system, to an outside organizer. The EQA organizer (also provider) provides a disclosed sample to a particular laboratory, this sample is sufficiently similar and homogenous to the biological sample and its properties or analyte concentration is known to the provider. The participating laboratory(ies) analyzes the sample and their results are compared with the true results from the EQA provider.

**Laboratory documents:** safety manuals, quality manuals, IQC records and EQA records, ensure in respective, safety in the laboratory and provision of quality laboratory services. Therefore, lack of these documents in a particular laboratory put its safety and quality into a question. Despite having four years of WHO-SLIPTA scheme in Tanzania, more than two third of district laboratories attained no star and were not registered for WHO-SLIPTA. This indicates poor preparedness of these laboratories towards ISO 15189: 2012 accreditation scheme. Report by Andiric *et al*. [10] documented that, lack of onsite training and mentorship; poor communications between laboratory management and other laboratory staffs; poor infrastructures; poor remuneration resulting to poor commitment; and selection of top management leaders (laboratory managers, quality officers and safety officers) based on seniority and not one´s demonstrated good performance are among challenges opposing the implementation of improvement towards accreditation among laboratories in sub-Saharan Africa.

**Study limitations:** limited funds constrained authors from enrolling clinical laboratories in all the Lake Zone Districts.

## Conclusion

In this study we observed that more than three thirds of district clinical laboratories affiliated to district and council designated hospitals in Lake Zone were not registered for WHO-SLIPTA indicating unpreparedness towards ISO 15189: 2012 accreditation. We therefore recommend to the Ministry of Health in Tanzania (MoHCDEC) through other collaborators and stakeholders to facilitate and support registration of district laboratories to WHO-SLIPTA for effective preparations towards ISO 15189: 2012 accreditation which ensures not only accurate and reliable results but also results are within turn-around-time.

### What is known about this topic

- The quality of clinical laboratory services pre-determine the quality of patients´ management provided by healthcare facility;
- Patients care (i.e., diagnosis of diseases) and public health (i.e., prevention and surveillance of outbreaks) is centered in quality services provided by health laboratories.

### What this study adds

- Most clinical laboratories in this study reported machinery breakdown and reagents out-of-stock due to lacking of machinery planned preventive maintenance and delayed supply of reagents respectively resulting in termination of services and direct impact on patient´s management;
- More than three thirds of district clinical laboratories affiliated to district and council designated hospitals at this setting were not registered for WHO-SLIPTA indicating unpreparedness towards ISO 15189: 2012 accreditation.
